# A Sliding‐Kernel Computation‐In‐Memory Architecture for Convolutional Neural Network

**DOI:** 10.1002/advs.202407440

**Published:** 2024-10-22

**Authors:** Yushen Hu, Xinying Xie, Tengteng Lei, Runxiao Shi, Man Wong

**Affiliations:** ^1^ State Key Laboratory of Advanced Displays and Optoelectronics Technologies Department of Electronic and Computer Engineering The Hong Kong University of Science and Technology (HKUST) Hong Kong China; ^2^ Guangzhou HKUST Fok Ying Tung Graduate School Guangzhou 511458 China

**Keywords:** convolutional computing, convolutional neural network, metal‐oxide, neuromorphic computing, thin film transistor

## Abstract

Presently described is a sliding‐kernel computation‐in‐memory (SKCIM) architecture conceptually involving two overlapping layers of functional arrays, one containing memory elements and artificial synapses for neuromorphic computation, the other is used for storing and sliding convolutional kernel matrices. A low‐temperature metal‐oxide thin‐film transistor (TFT) technology capable of monolithically integrating single‐gate TFTs, dual‐gate TFTs, and memory capacitors is deployed for the construction of a physical SKCIM system. Exhibiting an 88% reduction in memory access operations compared to state‐of‐the‐art systems, a 32 × 32 SKCIM system is applied to execute common convolution tasks. A more involved demonstration is the application of a 5‐layer, SKCIM‐based convolutional neural network to the classification of the modified national institute of standards and technology (MNIST) dataset of handwritten numerals, achieving an accuracy rate of over 95%.

## Graphical abstract

The sharing of convolution kernels in convolutional operations creates a significant asymmetry between data and weight (kernel) volume. This study optimizes convolutional operations at the hardware level by utilizing threshold adjustments in dual‐gate thin‐film transistors (TFTs). Achieved is a reduction in memory access operations of up to 88% through data preloading and the sliding of small kernels.

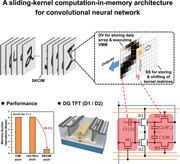

## 1 Introduction

**Figure 1:**
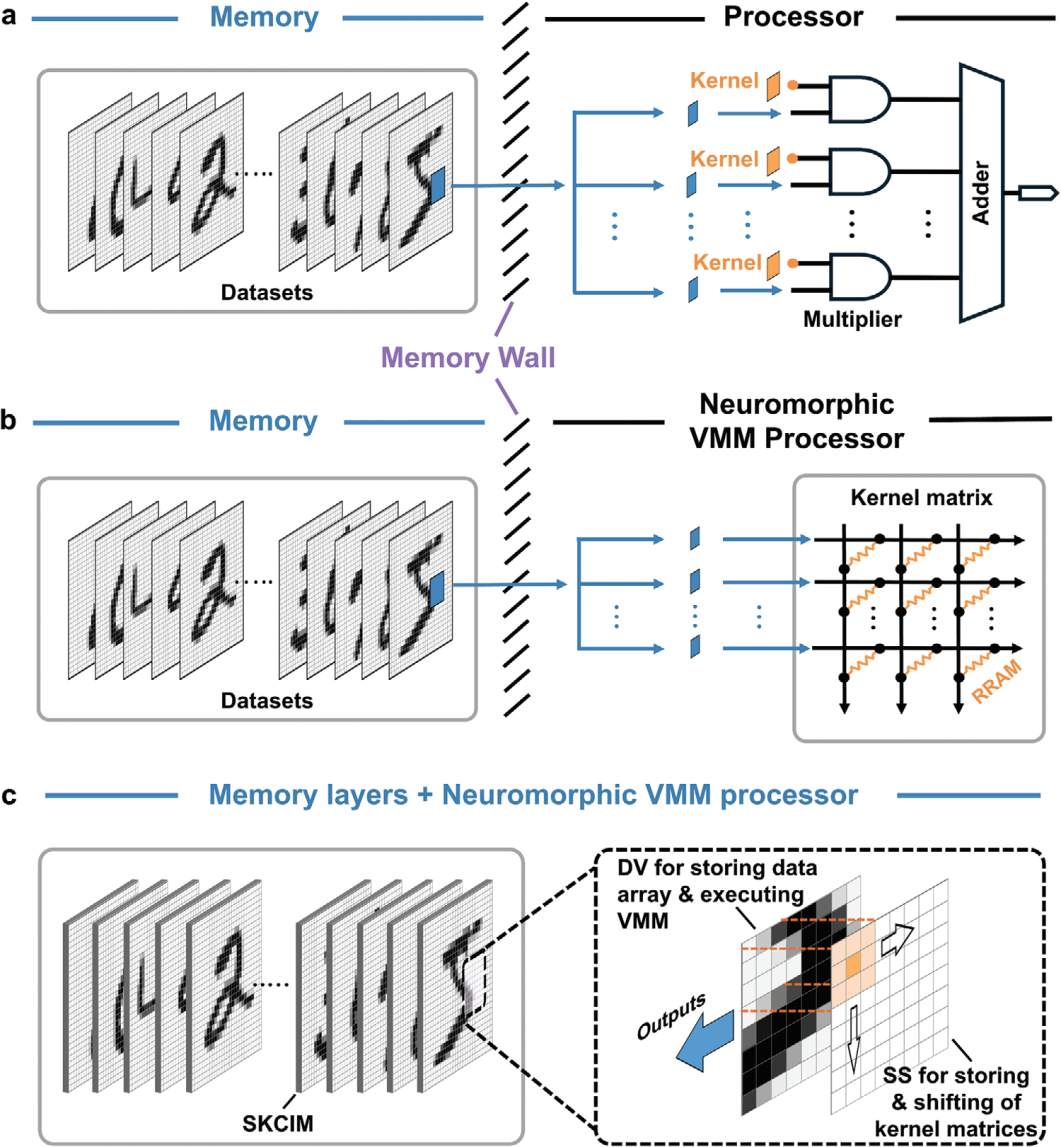
Hardware implementation of a CNN: a) von Neumann architecture, b) state‐of‐the‐art architecture with CIM‐based VMM processor, and c) the proposed SKCIM architecture.

Convolutional neural networks (CNNs)^[^ ^1^, ^2^ ^]^ for feature identification are extensively deployed in applications involving pattern recognition, such as face detection^[^ ^3^ ^]^ and autonomous driving.^[^ ^4^ ^]^ The convolution operation can be visualized as the “sliding” of a small kernel matrix across a typically much larger data array while executing neuromorphic computation involving “vector‐matrix multiplication” (VMM) of the overlapping elements of the kernel matrix and the data array.^[^ ^5^ ^]^ The result produced at each step is stored in a convolution feature map. When a CNN is implemented on a von Neumann computing machine with separate processor and memory units,^[^ ^6^, ^7^ ^]^ the small kernel matrix is held in the processor and repeated transfer of the elements of the data array from the memory to the processor and the elements of the feature map from the processor back to the memory is required while completing the convolution (**Figure** **1**a). Encountering what are often described as the energy and memory walls associated with neuromorphic computation,^[^ ^8^, ^9^ ^]^ the costs measured in energy and memory access time grow geometrically with increasing size of the data array.

Implementing computation‐in‐memory (CIM) processors^[^ ^10^, ^11^, ^12^, ^13^ ^]^ dedicated to speeding up VMM operations for convolution, device technologies for realizing the artificial synapses required for neuromorphic computation have been reported. These technologies are based on either nonvolatile or volatile memories. The former includes resistive random‐access memory (RRAM),^[^ ^14^, ^15^ ^]^ floating‐gate transistor memory (Flash),^[^ ^16^, ^17^ ^]^ ferroelectric random‐access memory (FeRAM),^[^ ^18^, ^19^ ^]^ phase change memory (PCM),^[^ ^20^ ^]^ and magnetoresistive random‐access memory (MRAM),^[^ ^21^ ^]^ while the latter encompasses charge‐based dynamic random‐access memory (DRAM),^[^ ^22^ ^]^ static random‐access memory (SRAM),^[^ ^23^ ^]^ and embedded DRAM (eDRAM).^[^ ^24^, ^25^ ^]^ For instance, Yao et al.^[^ ^26^ ^]^ employed a RRAM synaptic array, storing convolution kernels in the resistive elements and sequentially transferring blocks of the data array to the RRAM for computation, thereby implementing a CNN. This widely adopted scheme on CIM‐based VMM is well‐suited for fully connected (FC) neural networks, where the impact of transferring elements of the data array across the memory wall (Figure 1b) is negligible due to the size of input vector being significantly smaller than that of the weight matrix. By contrast, convolution operations involve numerous small matrix multiplications, and the substantial ratio of data volume to the size of the convolution kernels creates a notable distinction from conventional FC layer computations. Therefore, existing schemes for CIM‐based VMM for convolution calculations will exacerbate the previously encountered effects of transferring data array elements across the memory wall.

A more effective approach of overcoming the memory wall is to deploy CIM for executing VMM operations within the memory storing the data array, thus eliminating data transfer but demanding a mechanism for sliding a kernel matrix across the memory storing the data array. Presently proposed and displayed in Figure 1c is a “sliding kernel computation‐in‐memory” (SKCIM) system, which involves two overlapping layers of functional arrays, dubbed “DV” and “SS.” DV contains both memory elements for storing the data array and artificial synapses for neuromorphic VMM computation, whereas SS is used for storing and sliding the kernel matrices by sequentially erasing and writing of the components of a kernel matrix in the memory elements of SS. The frequent write–erase operations of convolution kernels present significant challenges to typical nonvolatile memory elements employed in the implementation of VMM. Specifically, RRAM and Flash suffer from low endurance and suboptimal write/read speeds; PCM exhibits deficiencies in both endurance and write power; while FeRAM and MRAM exhibit enhanced read/write capabilities and endurance, they encounter challenges related to cost and reliability.^[^ ^27^ ^]^ In contrast to nonvolatile memories, volatile memories such as DRAM, eDRAM, and SRAM typically offer superior read/write performance and endurance. Metal‐oxide (MO)‐based eDRAM capitalizes on the ultralow leakage current^[^ ^28^ ^]^ of MO thin‐film transistor (TFT) to achieve a pseudo‐nonvolatile storage behavior.

With performance fitting the purpose of electronic displays, MO TFTs have been commercially deployed on an industrial scale.^[^ ^29^, ^30^ ^]^ Enabled by their exceptionally low leakage current, they are being explored as switching elements for the construction of embedded memory^[^ ^31^ ^]^ based on capacitors as memory elements. Recently, Hu et al.^[^ ^32^ ^]^ reported artificial neural networks (ANNs) composed of arrays of dual‐gate (DG) TFTs as artificial synapses and capacitors as memory elements. A physical SKCIM system based on MO TFTs is presently described. A 5‐layer CNN image‐processing system is demonstrated, consisting of two pairs of convolutional and pooling layers, and one fully connected ANN output layer. All 5 layers are implemented using 32 × 32 SKCIM arrays. Combining in and ex situ training methodologies, the system achieves a prediction accuracy of 95% and a reduction of 88% in memory‐access operations when applied to classification of the modified national institute of standards and technology (MNIST) dataset of handwritten numerals. Theoretical estimation is given, comparing how the memory‐access operations scale with the sizes of the data array and the kernel matrix when executing CNN on a state‐of‐the‐art system with dedicated CIM‐based VMM processor or a SKCIM system.

### 1.1 Device Fabrication and Characterization

**Figure 2:**
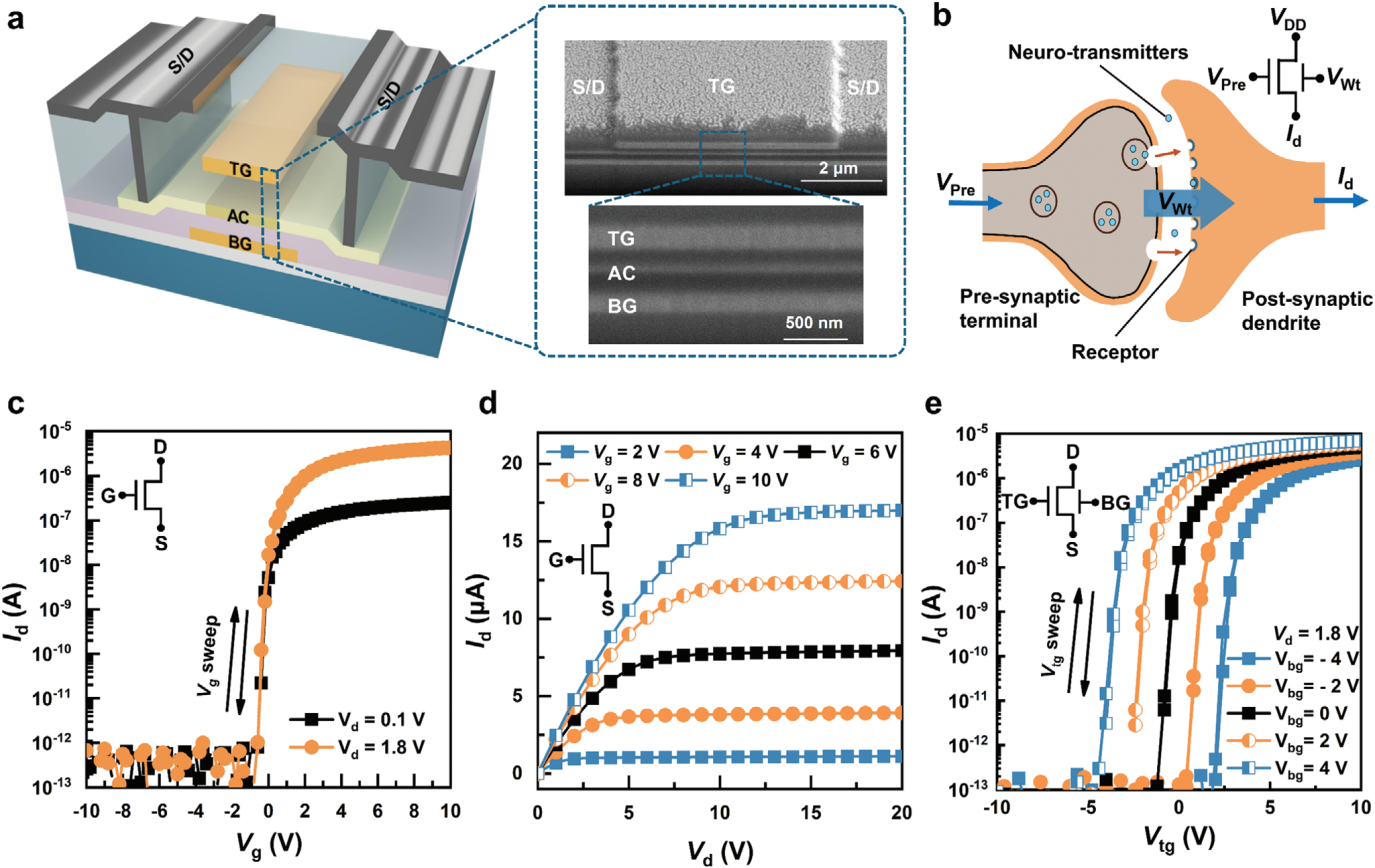
a) The 3D structural schematic and cross‐sectional SEM images of a DG TFT. b) A biological synapse and a DG TFT as its electronic counterpart. c) Transfer and d) output characteristics of a SG TFT. e) Transfer characteristics of a DG TFT.

A low‐temperature MO TFT technology^[^ ^33^, ^34^ ^]^ capable of monolithically integrating single‐gate (SG) TFTs, DG TFTs, and memory capacitors (see Device Fabrication subsection under the Experimental Section and Figure S1 in the Supporting Information) has been deployed for the construction of a SKCIM system. Shown in **Figure** **2**a are the schematic rendering and scanning electron microscopy (SEM) images of a DG TFT. With at least a portion of its active layer (AC) sandwiched between its bottom‐gate (BG) and top‐gate (TG) electrodes, a DG TFT as an artificial synapse^[^ ^35^, ^36^ ^]^ is illustrated in Figure 2b. Its drain current *I*_d_ emulating a postsynaptic current is simultaneously modulated by the presynaptic input (*V*_pre_) and the weight signals (*V*_Wt_) placed, respectively, on its high‐impedance TG and BG electrodes.

With the source voltage *V*_s_ of a SG TFT set to 0 V, the *I*_d_ versus gate voltage *V*_g_ transfer characteristics at drain voltage *V*_d_  =  0.1 and 1.8 V are shown in Figure 2c. The *I*_d_ versus *V*_d_ output characteristics at 5 values of *V*_g_ between 2 and 10 V are displayed in Figure 2d. The channel width (*W*) and length (*L*) of the TFTs are 5 µm. A threshold voltage *V*_T_ of −1.3 V and a field‐effect mobility of ≈15 cm^2^ V^−1^ s^−1^ are extracted from these characteristics. With the BG voltage *V*_bg_ set at various values between − 4 and 4 V, the *I*_d_ versus TG voltage *V*_tg_ transfer characteristics of a DG TFT presented in Figure 2e clearly exhibit simultaneous modulation of *I*_d_ by *V*_bg_ and *V*_tg_. The DG TFTs exhibit negligible threshold drift in their transfer characteristics across a range of operating temperatures, thereby demonstrating its exceptional thermal stability (Figure S2, Supporting Information). The uniformity of the TFTs has been characterized and shown in Figure S3 (Supporting Information).

### 1.2 The SKCIM Architecture

**Figure 3:**
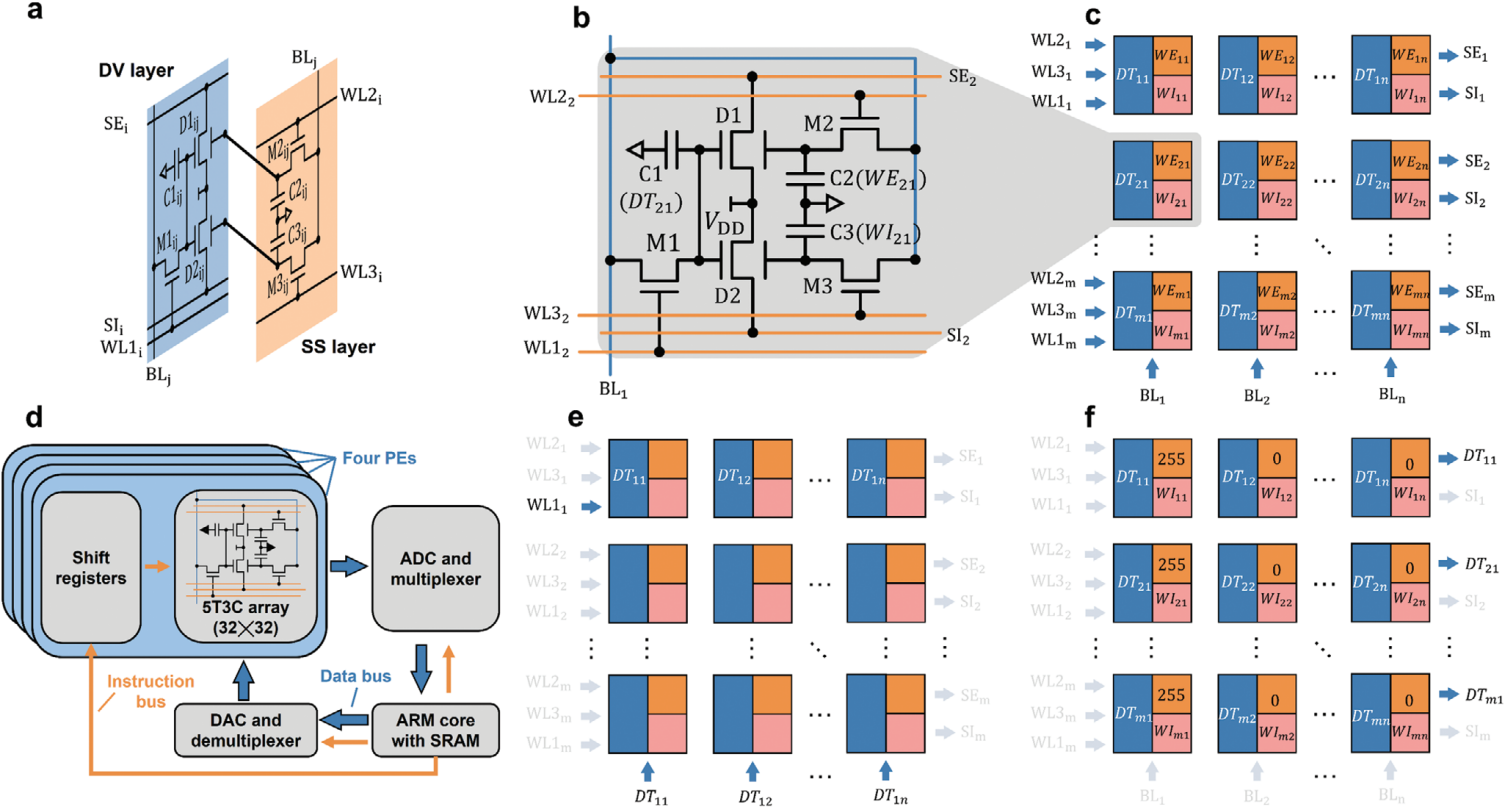
a) Two layers of a 5T3C cell. b) Schematic circuit diagram of a 5T3C cell, including 2 DG TFT artificial synapses, 3 memory capacitors, and 3 memory‐access SG TFTs. c) Schematic diagram of an *m*  ×  *n* circuit array based on 5T3C cells. d) Schematic diagram of the peripheral circuit used to drive the 5T3C array. Schematic diagram illustrating the principles of data e) writing in and f) reading from the 5T3C array.

Shown in **Figure** **3**a are the circuit schematics of cells making up the arrays in DV and SS. Each “3‐TFT‐1‐capacitor” (3T1C) cell at the *i*th $\left(i=1,\text{…},m\right)$ row and *j*th $\left(j=1,\text{…},n\right)$ column of an *m*  ×  *n* DV array consists of a capacitor memory element C1_*ij*_ for storing a component DT_*ij*_ of the data array, a SG TFT M1_*ij*_ for controlling access to the storage electrode of C1_*ij*_, and two DG TFTs D1_*ij*_ and D2_*ij*_ as artificial synapses for neuromorphic computation. M1_*ij*_ is switched using a control signal placed on the row‐wise word line WL1_*i*_ to store on C1_*ij*_ the signal placed on the column‐wise bit line BL_*j*_. The TG electrodes of D1_*ij*_ and D2_*ij*_ are biased at DT_*ij*_ stored on C1_*ij*_. The *I*_d_s of D1_*ij*_ and D2_*ij*_ are accumulated, respectively, on the row‐wise “excitatory” and “inhibitory” source lines SE_*i*_ and SI_*i*_.

The corresponding “2T2C” cell making up the SS array consists of two capacitor memory elements C2_*ij*_ and C3_*ij*_ for storing the respective excitatory and inhibitory weight signals WE_*ij*_ and WI_*ij*_, and two SG TFTs M2_*ij*_ and M3_*ij*_ for controlling access to the respective storage electrodes of C2_*ij*_ and C3_*ij*_. M2_*ij*_ and M3_*ij*_ are switched using the control signals placed on the respective row‐wise word lines WL2_*i*_ and WL3_*i*_ to store the weight signals placed on the shared bit line BL_*j*_. The corresponding cells in DV and SS are coupled by connecting the BG electrodes of D1_*ij*_ and D2_*ij*_ of a DV cell to the respective storage electrodes of C2_*ij*_ and C3_*ij*_ of a SS cell. When physically implemented, the 2 cells are monolithically integrated in a 5T3C circuit, as depicted in Figure 3b. Shown in Figure 3c is a schematic array showing the signals DT_*ij*_, WE_*ij*_, and WI_*ij*_ stored in a SKCIM cell. Presented in Figure S4 (Supporting Information) are photographic images taken of a 5T3C array, a SG TFT, and a DG TFT. For the present demonstration, *m*  =  *n*  =  32 and the physical sizes of the various components are summarized in Table S1 (Supporting Information).

A schematic block diagram of the peripheral system for operating a SKCIM system is depicted in Figure 3d. The system consists of four VMM processing elements (PEs), each based on a 32 × 32 array of 1024 5T3C cells and three shift registers. Photographs of the experimental setup are shown in Figure S5 (Supporting Information). Data and weight signals up to 8 bits resolution are generated using an ARM microprocessor run at a clock frequency of 168 MHz. They are converted to their analog counterparts DT_*ij*_, WE_*ij*_, and WI_*ij*_ using digital‐to‐analog converters (DACs) and distributed on the bit lines BL_*j*_ using demultiplexers. Shift registers are connected to the word lines WL1_*i*_, WL2_*i*_, and WL3_*i*_ to sequentially turn on the respective access SG TFTs M1_*ij*_, M2_*ij*_, and M3_*ij*_, thus passing the signals on BL_*j*_ to the respective memory capacitors C1_*ij*_, C2_*ij*_, and C3_*ij*_. Respectively, using transimpedance circuits and analog‐to‐digital converters (ADCs), the accumulated current signals on the source lines SE_*i*_ and SI_*i*_ are converted to a pair of voltage signals and digitized (Figure S6, Supporting Information). The resulting signals are selected using multiplexers and passed on to the microprocessor for the computation of a difference voltage.

The active‐matrix row‐scanning scheme deployed for placing DT_*ij*_, WE_*ij*_, or WI_*ij*_ on C1_*ij*_, C2_*ij*_, or C3_*ij*_ by sequentially turning on a row of M1_*ij*_, M2_*ij*_, or M3_*ij*_ using control signals placed on the word lines WL1_*i*_, WL2_*i*_, or WL3_*i*_ is schematically illustrated in Figure 3e for DT_*ij*_. The sequential scanning stops when the entire set of signals is loaded. The reading of the data stored on C1_*ij*_ is accomplished using active‐matrix column scanning, as demonstrated in Figure 3f. Except for one selected column, the D1_*ij*_s in the DV array are turned off by loading 0 V on the C2_*ij*_s. The *I*_d_ of the D1_*ij*_s along the selected column are available on the source lines SE_*i*_. The corresponding data stored on C1_*ij*_ can be inferred using a measured DT versus *I*_d_ calibration curve (Figure S7, Supporting Information). The sequential scanning stops when the entire set of DT_*ij*_ is read. Reading after writing of DT allows characterization of the storage performance of a capacitor memory element.

### 1.3 Long‐Term Memory Characterization of a 5T3C Cell

**Figure 4:**
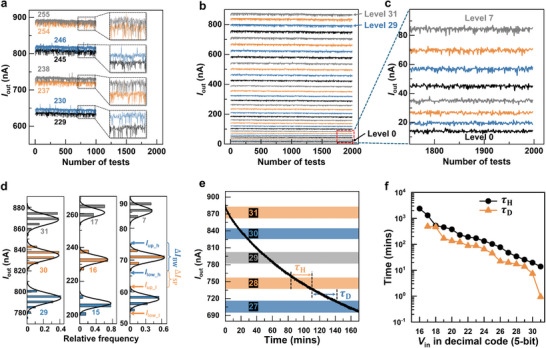
*I*_out_ versus *V*_in_ over 1000–2000 test cycles. a) Partially overlapping *I*_out_s of adjacent pairs of *V*_in_ in 8 bits resolution. b) Clearly resolved *I*_out_s of pairs of adjacent levels of *V*_in_ in 5 bits resolution even down to c) the lowest pair of adjacent Levels 0 and 1. d) Statistical distribution of the *I*_out_s of 3 groups of 3 consecutive levels of *V*_in_ in decimal codes. e) Time decay of *I*_out_ starting from Level 31 when the address TFT M1 is switched off. Hold (τ_H_) and decay (τ_D_) times are schematically indicated. f) Measured τ_H_ and τ_D_ corresponding to *V*_in_ in decimal codes of 16–31.

The same test circuit of Figure S3a (Supporting Information) was characterized using the custom‐built measurement system shown in Figure S5 (Supporting Information), with *V*_bg_, *V*_SS_, and *V*_DD_, respectively, set at 5, 3.2, and 5 V. An 8 bits input *V*_in_ between 1.8 and 5 V was fed on the bit line through a DAC to generate the *V*_BL_ to be written as *V*_tg_ on capacitor C1. The *I*_d_ of TFT D1 was converted to a voltage using a transimpedance circuit and digitized using an ADC. The output of the ADC is converted to a current *I*_out_ using a 1 MΩ load resistor R_E*i*_ or R_I*i*_ (Figure S6, Supporting Information). More than 2000 scans of the *I*_out_ versus *V*_in_ characteristics were recorded, and a representative set is shown in **Figure** **4**a. It is clear *I*_out_ could not be resolved with the same precision as *V*_in_, due to the significant overlap of the *I*_out_ corresponding to adjacent levels of *V*_in_. The scans were repeated with a 5 bits *V*_in_, with Levels 0 and 31 corresponding, respectively, to 1.8 and 5 V. It can be seen from the dependence shown in Figure 4b,c that *I*_out_ could be clearly resolved for all levels of *V*_in_. This reduction in precision from 8 to 5 bits is caused by the higher noise interference of the system compared to that of probe station.

Extracted from the 2000 scans and shown in Figure 4d are the distribution of *I*_out_ obtained at 9 levels of *V*_in_ corresponding to decimal codes of 31, 30, 29; 17, 16, 15; and 7, 6, 5. For each level of *V*_in_, one can extract an upper bound *I*_up_ and a lower bound *I*_low_ of its corresponding *I*_out_ distribution and compute a bandwidth Δ*I*_BW_  ≡  *I*_up_  −  *I*_low_; between any two adjacent levels, one extracts a lower bound $I_{\mathrm{low}\_h}$ of the higher level and an upper bound $I_{\mathrm{up}\_l}$ of the lower level and computes a band separation $\Delta I_{\mathrm{SP}}\;\equiv \;I_{\mathrm{low}\_h}-I_{\mathrm{up}\_l}$. When a *V*_in_ corresponding to Level 31 is stored on C1 and TFT M1 is turned off, the resulting *V*_tg_ (hence *I*_out_) decays due to leakage to Level 27 in about 170 min. The time dependence of *I*_out_ is exhibited in Figure 4e. The time it takes to traverse the Δ*I*_BW_ of a given level is defined as the “hold time” τ_H_ and the time it takes to traverse the Δ*I*_SP_ between adjacent bands is defined as the “decay time” τ_D_. The dependence of τ_H_ and τ_D_ on *V*_in_ are extracted and shown in Figure 4f. τ_H_ is shorter at a higher level of *V*_in_ and it is about 15 min at Level 31. This is sufficiently long for completing the convolution tasks in the present study.

The three capacitors in the 5T3C cell, in conjunction with the addressing transistors, enable long‐term signal storage. The two DG TFTs perform computations that emulate the behavior of synapses, including potentiation, depression, and both excitatory and inhibitory actions (see Characterization of Excitatory, Inhibitory, Potentiation, and Depression Behavior subsection in the Experimental Section and Figure S8 in the Supporting Information).

### 1.4 Operation of the SKCIM PEs

**Figure 5:**
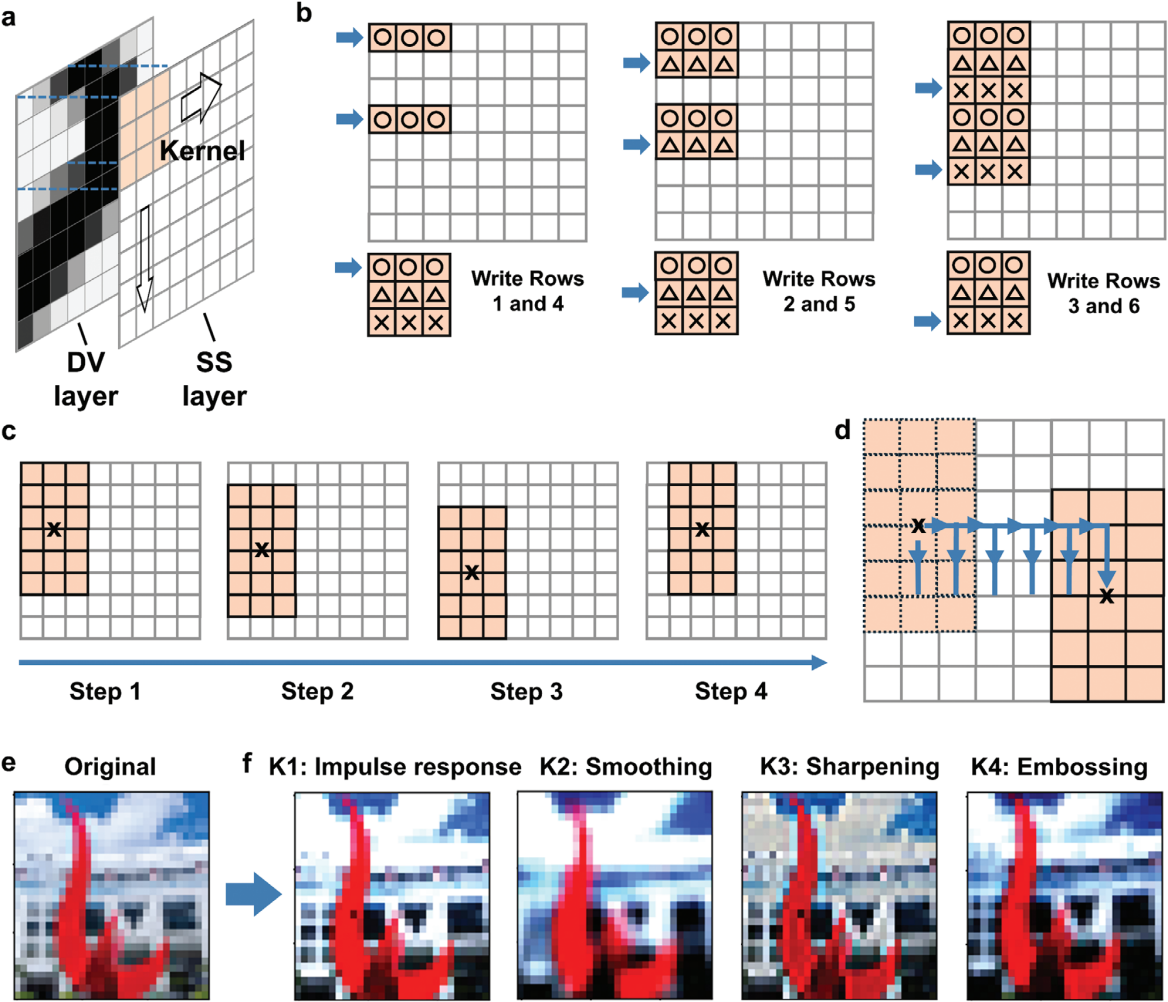
a) Schematic illustration of the storage of a data array in a DV layer and the “sliding” of a kernel matrix in a SS layer. b) Writing of two kernel matrices in an 8  ×  8 SS array, starting with their first rows. c) Schematic illustration of the simultaneous “sliding” of two kernel matrices. d) Schematic trajectory of the sliding of two kernel matrices in the SS layer. e) Original image. f) Examples of convolutional operations: impulse response, smoothing, sharpening, and embossing.

The sliding of a kernel matrix in the SS layer during convolution is schematically illustrated in **Figure** **5**a, using an 8  ×  8 data array and a 3  ×  3 kernel matrix as an example. The entire set of DT_*ij*_ is first loaded in the DV array; the WE_*ij*_ and WI_*ij*_ on C2_*ij*_ and C3_*ij*_ are initialized to 0 V, thus turning off all D1_*ij*_ and D2_*ij*_. Since 3 is not a factor of 8, only two 3  ×  3 kernel matrices can be stacked in an array with 8 rows. The operation starts with the sequential loading of the first, second, and last rows of the kernel matrix, respectively, in Rows 1 and 4, 2 and 5, and 3 and 6 of the SS array. Consequently, a 6  ×  3 portion of Columns 1, 2, and 3 of the SS array, containing 2 copies of the kernel matrices, is created (Figure 5b).

For each copy of the kernel matrix, the difference voltage values from the three rows corresponding to a kernel matrix are summed to generate a convolution feature value. In the next step of the operation, the vertically stacked 6  ×  3 kernel matrices are shifted down by 1 row, thus occupying Rows 2–7 of the SS layer. The respective WE_*ij*_ and WI_*ij*_ on C2_*ij*_ and C3_*ij*_ of Row 1 of the SS layer are reset to 0 V and a new pair of feature values are computed and recorded in their corresponding locations of the convolution feature map. This vertical sliding operation continues until the bottom edge of the SS array is reached. In the present example, 6 convolution feature values are generated and recorded. The respective WE_*ij*_ and WI_*ij*_ on C2_*ij*_ and C3_*ij*_ of the SS layer are reset to 0 V before the sequence of vertical sliding operations is repeated after laterally sliding the kernel matrices to the right by 1 column, now involving Columns 2, 3, and 4 (Figure 5c). This combination of lateral followed by vertical sliding operations continues until the right edge of the SS array is reached and a 6  ×  6 convolution feature map is obtained (Figure 5d). This SKCIM‐based implementation of CNN can be readily generalized to the combination of any *m*  ×  *n* data array and any *p*  ×  *p* kernel matrix.

Four PEs were selected to demonstrate the implementation of a CNN. The global uniformity characterization of each PE, along with the periodic scanning of PE1, is presented in Figure S9 (Supporting Information) (see Stability and Uniformity Characterization of SKCIM PEs subsection in the Experimental Section and Figure S9 in the Supporting Information). Exhibiting the best uniformity, PE1 is chosen for the demonstration of the convolution operations.

The utility of a SKCIM system for convolution is verified using a 30  ×  30 image (Figure 5e), with each pixel in the image consisting of 3 subpixels of the primary colors of “red,” “green,” and “blue.” Four convolution kernels K1, K2, K3, and K4 (Figure S10, Supporting Information) are deployed, producing convoluted images showing the respective effects of “K1: impulse response” for largely preserving the original image, “K2: smoothing” for blurring noise and details in the image, “K3: sharpening” for enhancing details and edges, and “K4: embossing” for directional edge enhancement (Figure 5f).

### 1.5 Comparison of von Neumann, State‐of‐the‐Art VMM Processor and SKCIM

**Figure 6:**
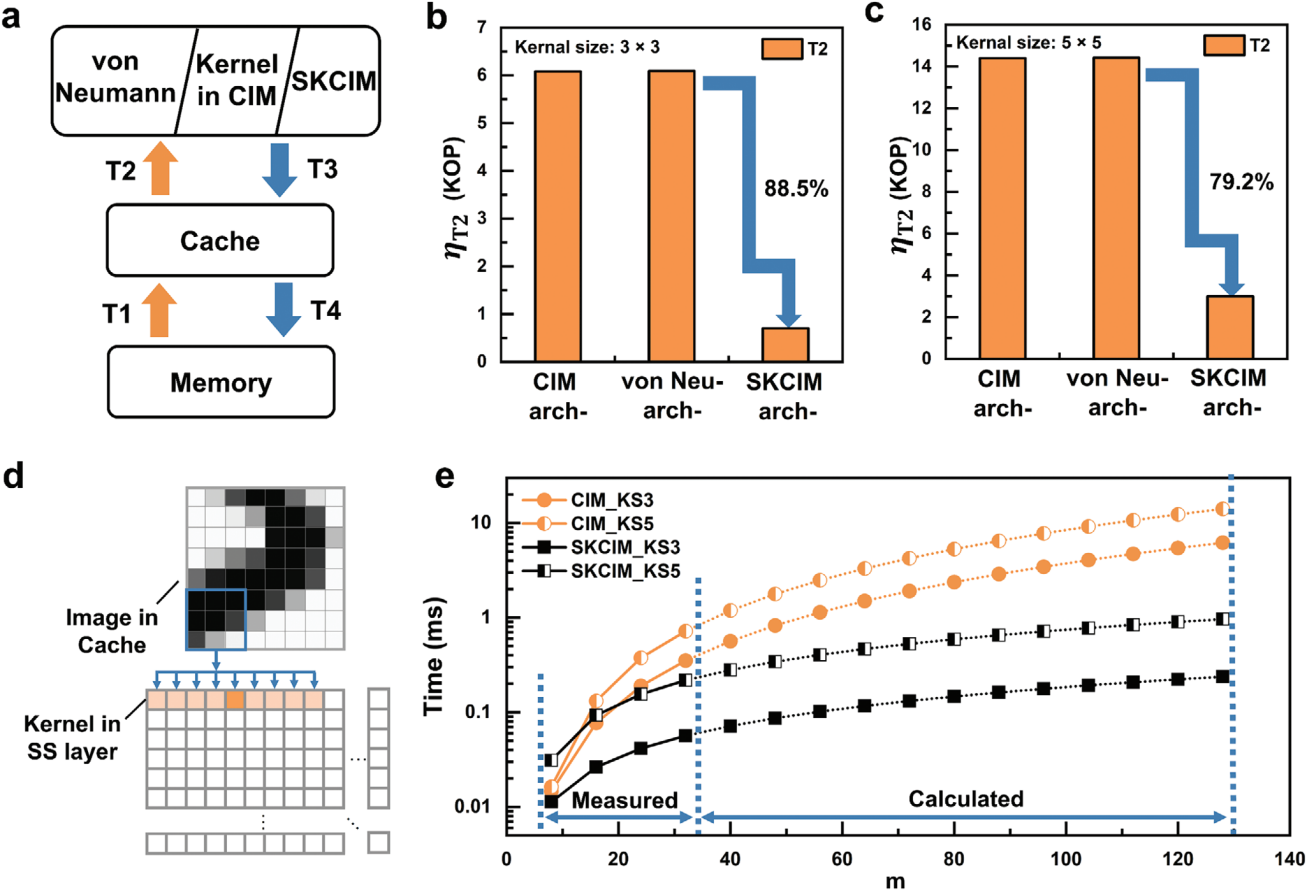
a) Schematic illustration of data transfer channels of T1–T4. Theoretical η_T2_ in kilo‐operations (KOP) of the three architectures with kernel matrix sizes of b) 3  ×  3 and c) 5  ×  5. d) Emulation of state‐of‐the‐art CIM‐based VMM system using a SKCIM system. e) Measured (solid line) and extrapolated (dotted line) time to complete η_T2_ data transfers of a state‐of‐the‐art CIM‐based VMM and a SKCIM system when executing convolution of a 28  ×  28 image with different kernel matrix sizes.

The three components of main memory, cache memory, and the processor required for convolution are considered. The processor is either a conventional processor for a von Neuman machine or a CIM‐based VMM processor. The corresponding channels (**Figure** **6**a) of memory‐access operations (MAO) are T1 for loading of the data array from the main memory to the cache, T2 for sequential loading of the components of the data array or the kernel matrix from the cache to the processor, T3 for transferring components of the convolution feature map from the processor to the cache, and T4 for saving of the feature map from the cache to the main memory.

Since T1, T3, and T4 are deployed in all three architectures of von Neumann, state‐of‐the‐art VMM processor, and SKCIM, comparison will be made only of the MAO associated with T2. Consider as an example an *m*  ×  *m* data array convoluting with a *p*  ×  *p* kernel matrix and producing a (*m*  −  *p*  +  1)  ×  (*m*  −  *p*  +  1) feature map. The number of MAO is *m* ^2^ for T1, and (*m*  −  *p*  +  1)^2^ for T3 and T4. For T2 of the first two architectures, the data array is divided into *p*  ×  *p* component blocks, each with *p* ^2^ elements. Since there are (*m*  −  *p*  +  1)^2^ blocks that need to be transferred from the cache to the processor, the resulting number of MAO is η_T2_  =  (*m*  −  *p*  +  1)^2^  ·  *p* ^2^. Note that η_T2_ changes quadratically with *m* when *m*  ≫  *p*. For the SKCIM architecture, since it is the elements of the kernel matrix rather than those of the data array that need to be transferred, there are (*m*  −  *p*  +  1) lateral sliding operations and *p* vertical sliding operations of *p* ^2^ elements of the kernel matrix. The product of the three terms gives rise to an η_T2_  =  (*m*  −  *p*  +  1)  ·  *p* ^3^. Note that η_T2_ changes linearly with *m* when *m*  ≫  *p*. Compared in Figure 6b,c are the η_T2_ of the three architectures for *m*  =  28 and *p*  =  3 and 5, respectively. It can be seen that η_T2_ for the SKCIM architecture is significantly reduced compared to those for the other two architectures, respectively, by 88% and 79% for *p*  =  3 and 5.

The state‐of‐the‐art VMM processor for implementing convolution can be emulated using the SKCIM system (Figure 3d), as illustrated in Figure 6d. In this system, SKCIM‐based convolution is performed by storing data in the DV layer of the 5T3C array while sliding the convolution kernel across the SS layer. Whereas the 5T3C array emulates the state‐of‐the‐art VMM processor for convolution calculations by positioning the unfolded convolution kernel in the first row of the SS layer and transferring image data from the cache in blocks to the first column of the DV layer for computation with the SS layer. For example, A *p*  ×  *p* kernel matrix is flattened and loaded on the weight‐storing capacitors in the first row of the SS array. The M1_*ij*_s are turned on and *p*  ×  *p* blocks of the data array to be convoluted is prepared and sequentially moved to the first row of the DV array for computation. Presented in Figure 6e is the measured data transfer time from the cache to the processor to complete a convolution. For both *p*  =  3 and 5, the transfer times are measured for *m*  =  8, 16, 24, and 32. η_T2_ is used to estimate the number of transfer operations and applied to extrapolate the total transfer times for data array with *m*  >  32 and up to 128. Both emulated state‐of‐the‐art VMM processor and SKCIM are investigated. The latter exhibits obvious reduction in the required transfer time for a given *m*, consistent with the different dependences of η_T2_ on *m* for the two architectures.

### 1.6 Application of SKCIM to MNIST Recognition

**Figure 7:**
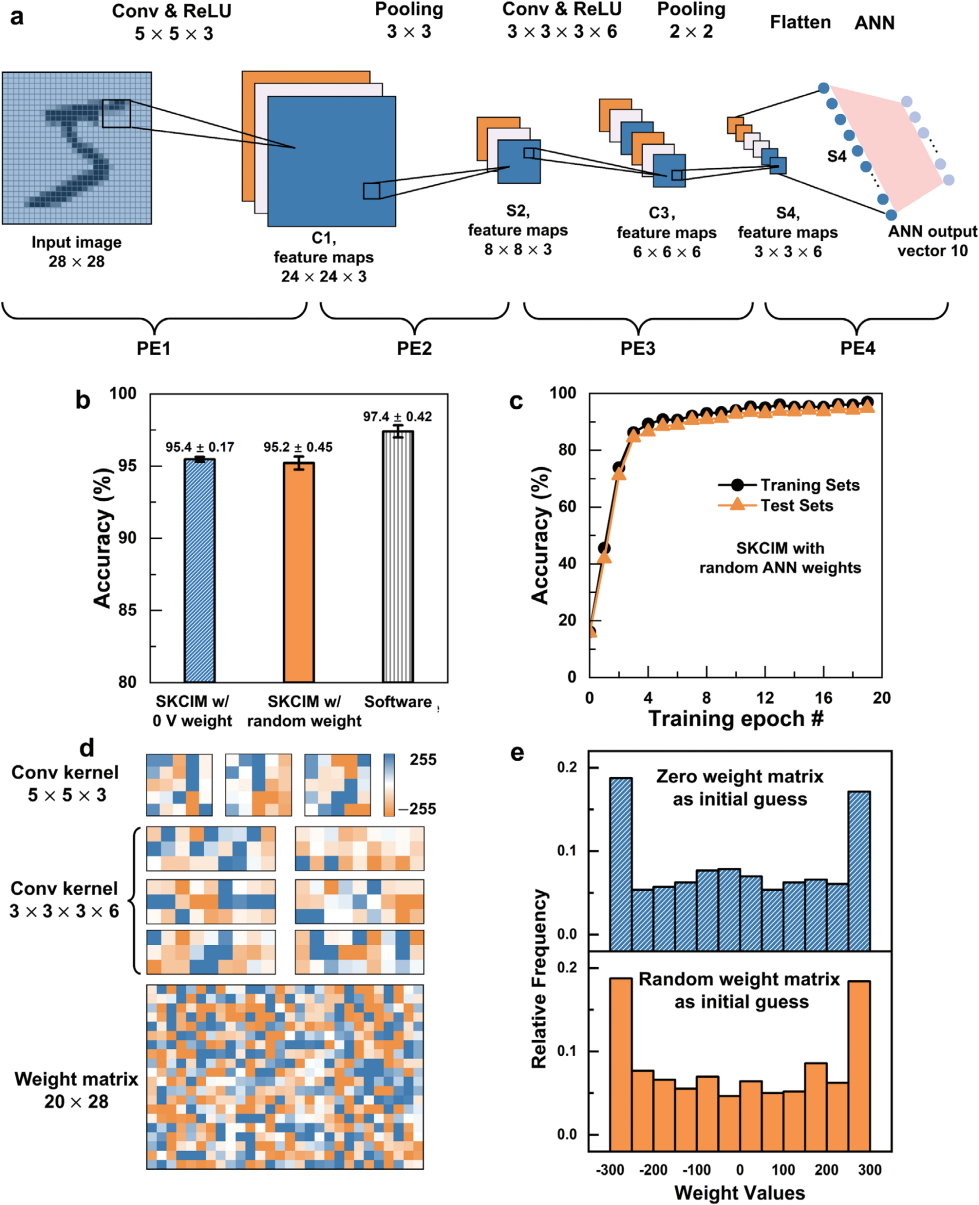
a) Structure of the 5‐layer CNN used for recognition of the MNIST dataset of handwritten numerals: 2 convolutional layers, 2 pooling layers, and a fully connected ANN layer. b) Comparison of the accuracy rates of the SKCIM systems with two different initial weight values and the software implementation. c) Evolution of the accuracy rates obtained using the training and the inference sets. d) Kernel matrices and ANN weight values after training. e) Statistical distribution of the weight values corresponding to initial conditions of zero and random weights.

Consisting of two pairs of convolution followed by pooling layers and a fully connected ANN output layer, a 5‐layer system (**Figure** **7**a) for classification of the MNIST dataset of handwritten numerals has been constructed. The layers are implemented using four SKCIM PEs. At 28  ×  28, the size of the image is smaller, thus it can be fully accommodated by a larger 32  ×  32 SKCIM PE. Three 5  ×  5 convolution kernel matrices are deployed to generate a 24  ×  24  ×  3 feature map C1 using the first layer. C1 is downsampled by pooling using a 3  ×  3 filter to generate an 8  ×  8  ×  3 feature map S2 using the second layer. Six 3  ×  3  ×  3 convolution kernel matrices are applied to S2 to generate a 6  ×  6  ×  6 feature map C3 using the third layer. Subsequently, C3 is downsampled by pooling using a 2  ×  2 filter to generate a 3  ×  3  ×  6 feature map S4 using the fourth layer. Finally, S4 is flattened and fed to the fully connected 56  ×  10 ANN output layer.

The implementation of the ANN layer using a 32  ×  32 SKCIM PE is accomplished by splitting the 54 inputs of the flattened P4 into two rows of 27 inputs. A 2  ×  28 input data array is generated after each row is augmented with a bias value. The first 20 rows of the SKCIM PE are used, with the first and second rows of the data array, respectively, loaded in the odd and even numbered rows of the SKCIM PE. Starting from the top of the SKCIM PE, the outputs of every consecutive pair of odd and even numbered rows are accumulated to generate a total of 10 outputs from the 20 rows of the ANN.

While the training of a CNN requires external computers for ex situ training to improve training speed, the inference relies entirely on hardware implementation with enhanced operational efficiency. A sequence of ex situ followed by in situ methodologies is adopted^[^ ^14^ ^]^ for training the SKCIM‐based physical system. The ex situ methodology involves the construction using Python and TensorFlow of an approximate software model that closely resembles the physical system. Rectified linear unit (ReLU) is used as an activation function. The convolutional kernel matrices and the weight parameters for the ANN are obtained. An accuracy of 97.4% (Figure 7b) is achieved on a test set comprising 10 000 samples using an optimally trained model. After appropriate scaling to match the electrical bias adopted to operate the physical system, the transformed kernel matrices are deployed for convolutional operations (see System Training subsection in the Experimental Section).

The weight parameters of the ANN were initialized either to 0 V or random values before in situ training of the ANN is carried out to account for the difference between the software and the physical systems, and to accommodate the nonuniformity in TFT parameters in the latter. An approximate gradient of the cost function is estimated and used to update the weight parameters. This process is repeated until convergence of the weight parameters is achieved. More details are given in Note S1 (Supporting Information).

Depicted in Figure 7c are the evolution of the inference accuracy versus training epochs for a system with kernel matrices obtained from ex situ training and ANN weights initialized to random values. Starting with a relatively low ≈15%, the predicted accuracy quickly saturates after 4 or 5 epochs of training and reaches ≈95% after 16 training epochs. Shown in Figure 7d are the kernel matrices and the trained ANN weights. As a comparison, the statistical distribution of the weights corresponding to initial ANN weights of 0 V and random values are shown in Figure 7e, showing largely similar trends.

Summarized in **Table** **1** is a comparison of the proposed SKCIM and other systems based both on TFTs and conventional Si transistors. For the present system implemented using a 5 µm indium–tin–zinc oxide (ITZO) TFT technology, a conservative working clock frequency of 1 MHz was used. The maximum number of parallel neuromorphic instructions computed per clock cycle is 32 × 32 × 2 = 2048, resulting in a maximum throughput of 8.192 giga operations (GOP) s^−1^ (Table S2, Supporting Information). Since the operating speed of a TFT scales with *L* ^−β^ for 1  ≤  β  ≤  2, the throughput could be increased by tenfold to 100‐fold when length *L* is scaled by tenfold from 5 to 0.5 µm.^[^ ^37^ ^]^ It should be noted that MO TFT with *L* below 10 nm^[^ ^24^ ^]^ and operating frequencies over GHz^[^ ^37^, ^38^ ^]^ have been reported.

**Table 1:** Comparison with other published SNNs.

	This work	IEEE Transactions on Circuits and Systems I ’23^[^ ^24^ ^]^		Japanese Journal of Applied Physics 20^[^ ^22^ ^]^	International Solid‐State Circuits Conference 21^[^ ^25^ ^]^	International Conference for High Performance Computing 23^[^ ^31^ ^]^
Technology	TFT 5 mm	TFT 45 nm	TFT 4 mm	TFT 350 nm/Si 110 nm	Si 65 nm	Si 40 nm
Frequency	1 MHz	N.A.	N.A.	25 MHz	50 MHz	N.A.
CIM scheme	eDRAM	eDRAM	eDRAM	eDRAM	DRAM	DRAM
Retention	≈40 h^a)^	20 s^b)^	≈10 h^b)^	>30 h^c)^	N.A.	N.A.
Precision [bit]	5	4	Analog	N.A.	8	8
Power	348 mW	N.A.	N.A.	9.95 mV	0.99 mW	1.06 W
Efficiency (Tera Operations Per Second W^−1^)	23.5^d)^	686^d)^	0.728^d)^	5	4.76	2.41

^a)^ Defined as median state degradation;

^b)^ Defined as the time at 0.5 least significant bit output error with evaluated number of rows in a multiply accumulate;

^c)^ Defined as 5% degradation for analog multiplication;

^d)^ CIM array only.

## 2 Conclusion

Consisting of two interconnected layers of memory arrays, a SKCIM architecture is proposed and demonstrated. One layer capable of in‐memory neuromorphic computation is used to store the data array; and the other is used to store and to shift the weight parameters. SKCIM can be applied to realize both convolutional and fully connected artificial neural networks. A MO TFT technology is used to physically implement a SKCIM system, allowing monolithic integration of the two layers in an array consisting of 5‐TFT‐3‐capacitor cells. The use of a capacitor as a memory element is made possible by the extremely low leakage current of a MO TFT. Dual‐gate TFT allowing simultaneous modulation of the channel current by its two separate gate biases is deployed as an artificial synapse for neuromorphic computation. Physical SKCIM systems have been constructed and deployed to execute convolution tasks and to classify the MNIST dataset of handwritten numerals.

## 3 Experimental Section

### Device Fabrication

The devices were fabricated on a 4 in. wafer covered with a buffer layer of 500 nm thick thermally grown silicon dioxide. Both the TG and the BG electrodes were deposited and patterned from a 100 nm thick layer of sputtered molybdenum (Mo). Formed above the BG was a gate insulator (GI) stack of 75 nm thick silicon oxide (SiO*_x_*) on top of a 50 nm thick layer of silicon nitride (SiN*_y_*), both deposited using plasma‐enhanced chemical vapor deposition (PECVD). The GI beneath the TG was made of a layer of patterned 100 nm thick PECVD SiO*_x_*. The active layer was formed of a 20 nm thick layer of sputtered ITZO. The conductive source and drain (S/D) regions were realized by subjecting the devices to an oxygen plasma treatment. Enveloping the device, a passivation layer of 250 nm thick SiO*_x_* was deposited. The S/D electrodes were composed of a stack of 300 nm thick aluminum on top of a 50 nm thick Mo. Single‐gate TFTs without BG electrodes were similarly constructed. The capacitors were fabricated from a combination of the BG, the SiO*_x_*/SiN*_y_* stack of GI, and ITZO conductive layers (Figure S1, Supporting Information).

### Stability and Uniformity Characterization of TFTs

A schematic of the circuit deployed for characterizing TFT stability and uniformity is shown in Figure S3a (Supporting Information). Measurements were made using Keysight B1500A semiconductor parameter analyzer. The respective control signals of *V*_WL_ =  0 and 5 V were applied to turn the address TFT M1 “off” and “on.” Due to the negative *V*_T_ of about −1.3 V (Figure 2c) of a TFT realized using the present technology, the minimum value of *V*_tg_ that could be stored on capacitor C1 must be sufficiently positive when *V*_WL_ =  0 V. Presently this was chosen to be 1.8 V to allow an adequate margin. Following the same consideration, *V*_SS_ =  3.2 V was chosen to ensure TFT D1 was turned off at *V*_bg_  =  *V*_tg_  =  1.8 V. With *V*_bg_ set at 4 different values of 2, 3, 4, and 5 V, the stability of the *I*_d_ versus *V*_tg_ transfer characteristics of a DG TFT at a supply voltage *V*_DD_ =  5 V was investigated by scanning *V*_tg_ in 256 steps (8 bits) from 1.8 to 5 V. Each step was identified by its decimal code, with 0 and 255 corresponding, respectively, to 1.8 and 5 V. More than 1000 characteristics were recorded, and those at the 1st, 140th, 540th, and 1040th scans are plotted in Figure S3b (Supporting Information). Their nice overlap at each given *V*_bg_ reflected a good measurement stability of the chosen TFT. The characteristics of 20 randomly selected TFTs are exhibited in Figure S3c (Supporting Information). Although variations were clearly exhibited among the TFTs, the inherent self‐compensation of an in‐situ training method (see the Experimental Section) was expected to mitigate the effects of TFT nonuniformity on the accuracy of neuromorphic computation.

### Characterization of Excitatory, Inhibitory, Potentiation, and Depression Behaviors

The results of a single 5T3C cell emulating biological synaptic behavior are illustrated in Figure S8a (Supporting Information). Weight signal Wt, ranging from −255 to 255 with a step interval of 51, were sequentially written into C2 and C3, as depicted in Figure 3b. The sign of Wt dictated whether the absolute value was assigned to C2 or C3; for instance, if Wt was −204, then WE_21_ = 0 and WI_21_ = 204; conversely, if the signal was positive, WI_21_ was set to 0. The input signal DT was incrementally scanned from 0 to 255, and the current outputs from D1 and D2 were recorded. The final current was derived by subtracting the output current of D2 from that of D1. Further analysis of Figure S8a (Supporting Information) indicated that when DT was fixed at 255 and scanning Wt from −255 to 255 yielded results reflecting the linearity and symmetry of the artificial synapse, as shown in Figure S8b (Supporting Information). It can be concluded from Figure S8 (Supporting Information) that the 5T3C cell demonstrated excellent symmetry due to the employed differential computation scheme. Furthermore, the threshold modulation functionality of the DG TFTs brought a potentially beneficial nonlinear behavior to the computational dynamics of the artificial synapse.

### Stability and Uniformity Characterization of SKCIM PEs

Presented in Figure 3f is a schematic representation of the data writing and reading process for the 5T3C array. The same method was employed to evaluate the uniformity of the four SKCIM PEs. A 32  ×  32 data matrix, with all values initialized to 255, was written to each SKCIM PE. Subsequently, for each column *j*, the excitatory weight WE_*j*_ was set to 255 and inhibitory weight WI_*j*_ was set to 0, while the excitatory weights for the remaining columns was maintained at 0. The difference between the SE_*i*_ and the SI_*i*_ for each row *i* was recorded as the scanning result for the cell at the intersection of the respective row *i* and the current scanning column *j*. This procedure was iteratively applied to the subsequent columns. The comprehensive global scanning results for each PE are illustrated in Figure S9a (Supporting Information). Utilizing the same scanning method, multiple repeat scans were performed on PE1, with a scanning interval of 0.5 s and a maximum of 100 000 scans (Figure S9b, Supporting Information).

### System Training

Ex situ training entailed constructing using Python and TensorFlow an approximate copy of the 5‐layer system on a computer for obtaining the convolution kernel matrices and the weight parameters by deploying the ReLU activation function and backpropagation (BP) technique. Training was completed within 20 epochs. The kernel matrices yielding the best results were selected. Their components were normalized using the Min‐Max Scaling method and adjusted to fit the decimal range of −255 to +255. After appropriate scaling to match the electrical bias adopted to operate the physical system, the kernel matrices with transformed parameters were deployed for convolutional operations during the subsequent round of in situ training deployed to address the approximate nature of the software model and accuracy loss resulting from inherent TFT nonuniformity.

While the kernel matrices were not modified during the in situ training, the weight parameters of the ANN layer were initialized and adjusted. For member *k* of a training set, the dependence of the output vector *Y_k_* on the input vector *X_k_* was approximated by

(1) $$Y_{k}\approx \mathrm{ReLU}\left(W^{T}\cdot X_{k}\right)$$

where *W* ^T^ is the transpose of the weight matrix *W*. A cost function *J* could be defined and computed over the *n* elements of the training set

(2) $$J\equiv \frac{1}{2n}\sum_{k=1}^{n}{\left(L_{k}-Y_{k}\right)}^{2}$$

where *L_k_* is the label output vector for *X_k_* and to which *Y_k_* should approach. Consequently, the gradient ∂*J*/∂*W* for BP could be computed according to

(3) $$\frac{\partial J}{\partial W}=\frac{\partial J}{\partial Y}\cdot \frac{\partial Y}{\partial W}\approx \left(L-Y\right)\cdot X^{T}$$

*W* was subsequently updated for the next epoch of training.

### Performance Characterization

For the 32  ×  32 SKCIM system with two DG‐TFT computation units per cell, the maximum throughput was determined by the product of the total number of operations (32  ×  32  ×  2) and the operating frequency. However, the limitations imposed by the process node (5 µm) and the relatively low mobility of a metal‐oxide TFT have led to a relatively conservative operating frequency of 1 MHz, resulting in a throughput of 8.192 GOP s^−1^.

### The maximum write energy *E* for each 5T3C cell was given by

(4) $$E=\frac{1}{2}C\left(V_{\max}^{2}-V_{\min}^{2}\right)$$

where *C* is the capacitance of a memory capacitor; *V*_max_ and *V*_min_ are the maximum and minimum data values.

The total power consumption *P* in the 5‐layer CNN contained the power consumption of each layer within the network. For each layer, the power consumption was calculated by multiplying the cumulative output currents from all SE and SI by the voltage applied to the DG TFTs. A total of 200 images were selected for each of the 10 unique digits in the MNIST dataset, and their corresponding power consumption *P* was computed and illustrated in Figure S11a (Supporting Information). The average power consumption was found to be 348 µW, as detailed in Table S2 (Supporting Information). Furthermore, this study presents the power consumption calculations for each layer in Figure S11b (Supporting Information).

### Data Acquisition

Capacitance measurements were carried out using a precision LCR meter (E4980A Agilent). The cross‐sectional SEM images of DG TFTs were acquired using the FEI Helios G4 UX. The transfer and output characteristics of the TFT were analyzed utilizing a probe station equipped with an Agilent 4156C.

## Conflict of Interest

The authors declare no conflict of interest.

## Author Contributions

Y.H. and M.W. designed the experiments. T.L. assisted Y.H. in device fabrication. X.X. proposed the process flow. R.S. collected and analyzed the data. All the authors discussed the results and contributed to the paper preparation. Y.H. and M.W. wrote the paper.

## Supporting information



Supporting Information

## Data Availability

The data that support the findings of this study are available in the supplementary material of this article.
