# Supplementary material for: A Sliding‐Kernel Computation‐In‐Memory Architecture for Convolutional Neural Network
Source: Adv Sci (Weinh). 2024 Oct 22;11(46):2407440. doi: 10.1002/advs.202407440 (PMC11633514; doi:10.1002/advs.202407440)
Supplement: Supplementary file 1 — Supporting Information [file ADVS-11-2407440-s001.pdf]

ADVANCED SCIENCE
Open Access

Supporting Information

for *Adv. Sci.*, DOI 10.1002/advs.202407440

A Sliding-Kernel Computation-In-Memory Architecture for Convolutional Neural Network

Yushen Hu, Xinying Xie, Tengteng Lei, Runxiao Shi* and Man Wong**

A sliding kernel computation in-memory architecture for convolutional neural network

Yushen Hu[1], Xinying Xie[1], Tengteng Lei[1], Runxiao Shi[1], Man Wong[1,2]

Corresponding author: eemwong@ust.hk, eershi@ust.hk, eetlei@ust.hk

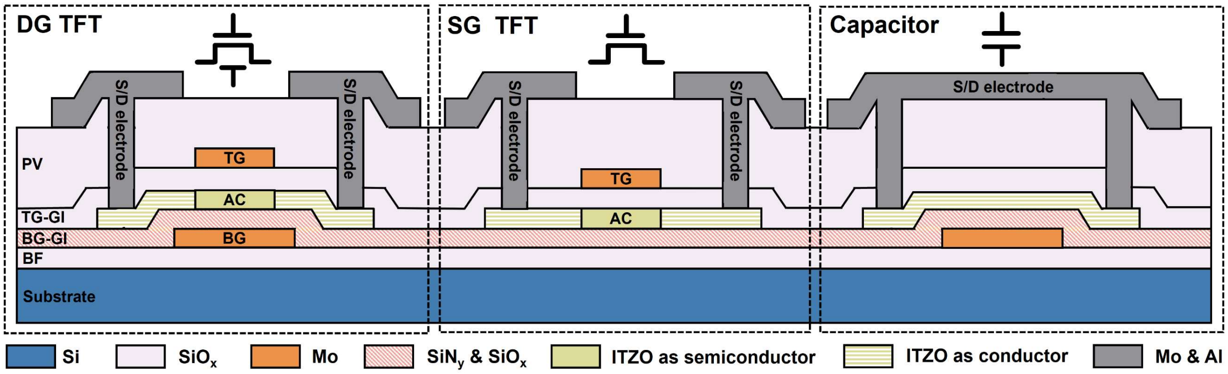

Fig. S1. Cross-sectional schematics of monolithically integrated DG TFT (left), SG TFT (middle) and capacitor (right).

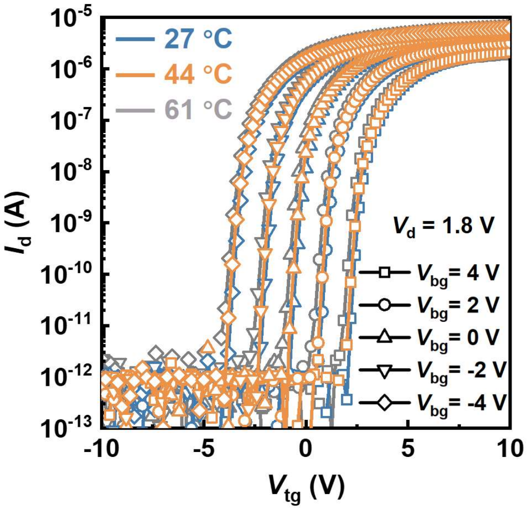

Fig. S2. Transfer characteristics of a DG TFT at three different temperatures.

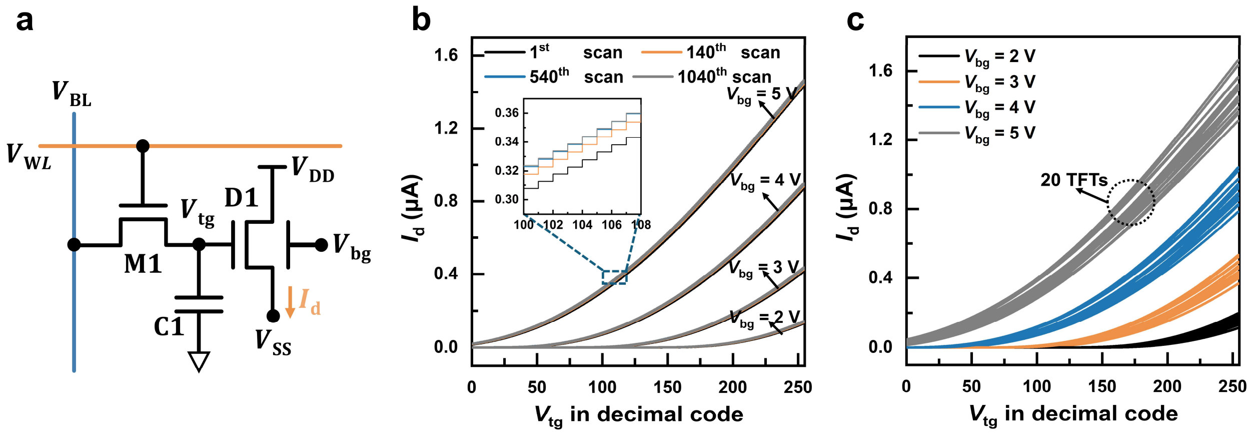

Fig. S3. (a) Schematic of the test circuit. (b) Stability test of a DG TFT D1 subjected to more than 1000 repeated scans. (c) Transfer characteristics of 20 randomly selected DG TFTs across a 4-inch silicon wafer, exhibiting statistical variations.

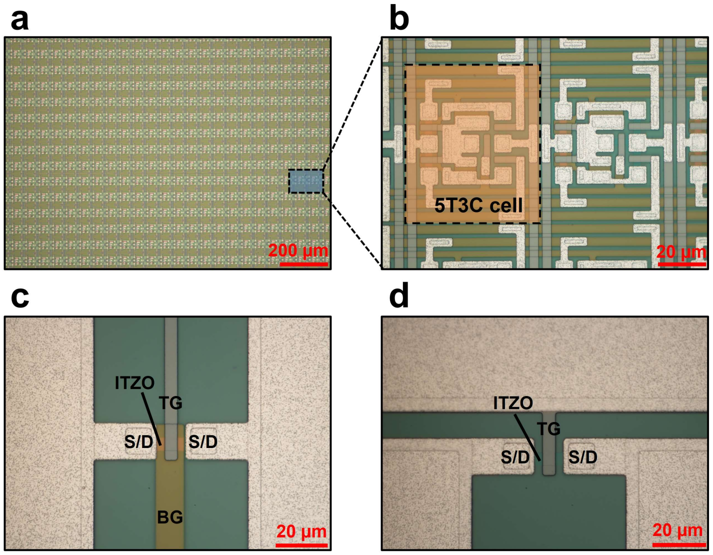

Fig. S4. (a) Magnified image of a SKCIM array. (b) Magnified 5T3C cell circuit. One DG TFT (c) and one SG TFT (d) included in the test structure, both of which have the same W/L of 5 μm/5 μm as the devices in the array circuit.

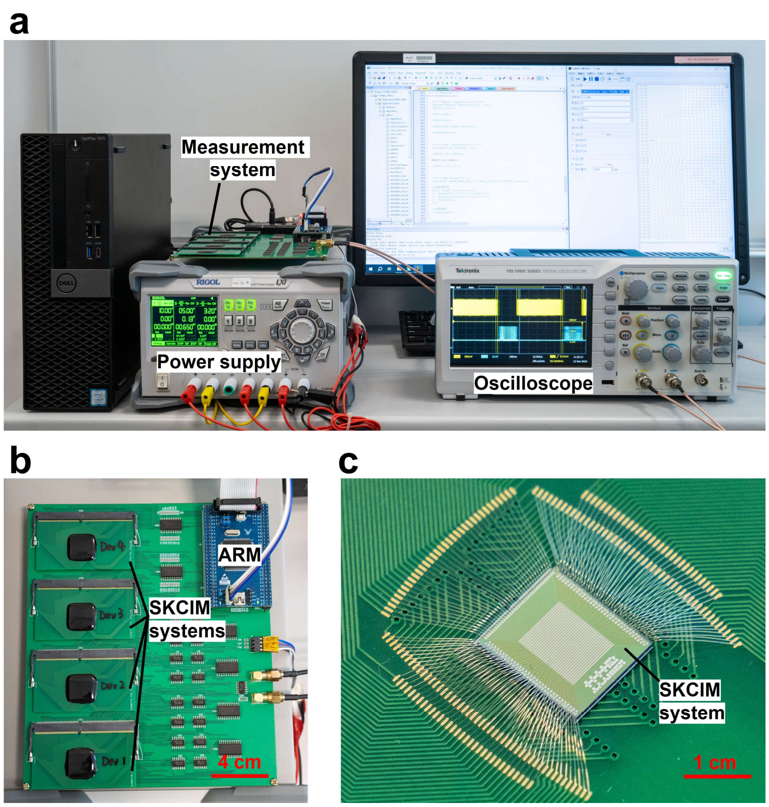

Fig. S5. Photographs of (a) test system, (b) peripheral circuit and (c) SKCIM system bonded on PCB.

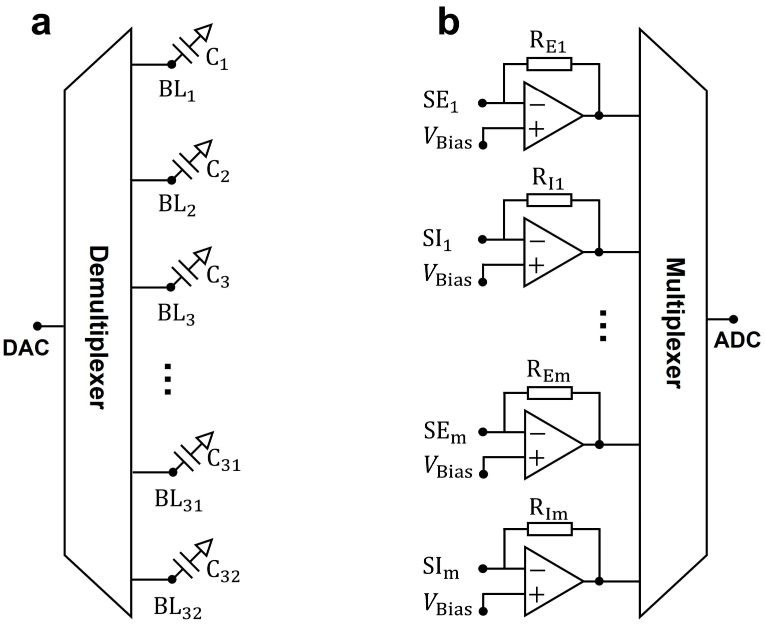

Fig. S6. Schematic diagrams of (a) data writing and (b) output sampling circuits.

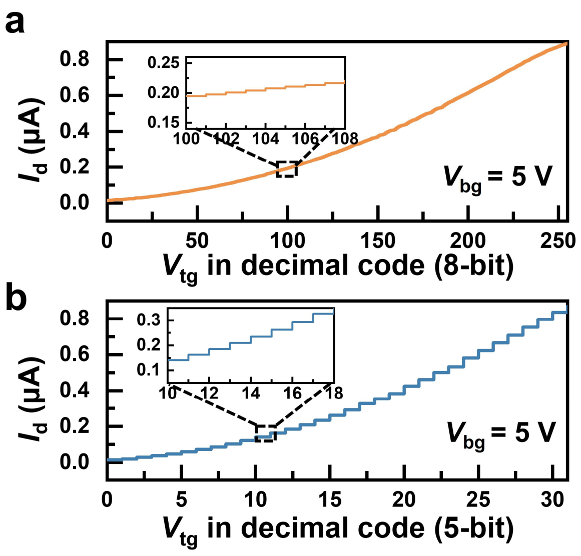

Fig. S7. Measured *DT* *vs*. I_d calibration curves with (a) 8-bit and (b) 5-bit resolution.

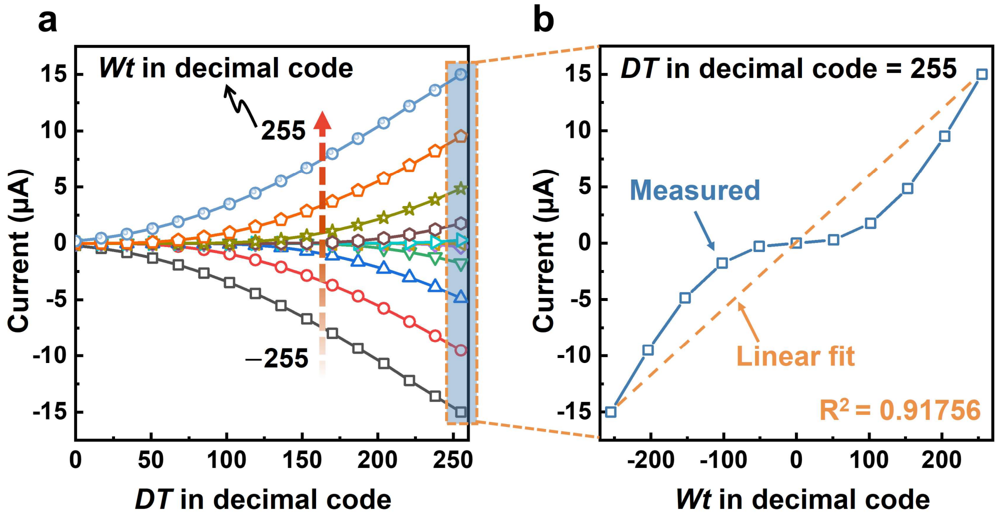

Fig. S8. (a) Characterization of excitatory, inhibitory, potentiation, and depression behavior of a single 5T3C cell. (b) Characterization of nonlinearity and asymmetry.

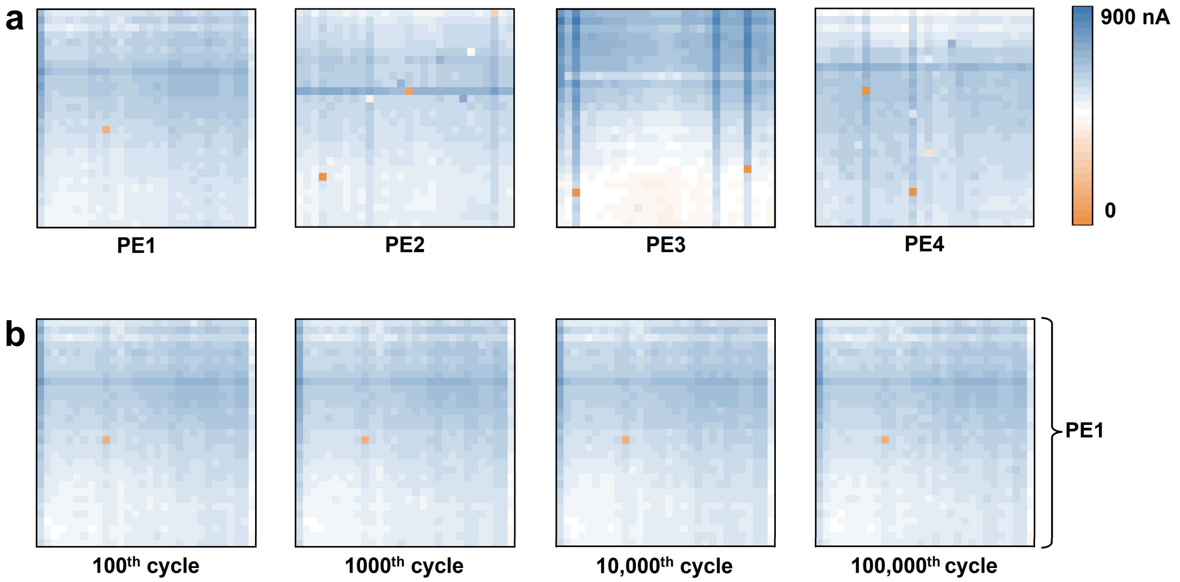

Fig. S9. Uniformity characterization: (a) device-to-device variation of four PEs, and (b) cycle-to-cycle variation of PE1.

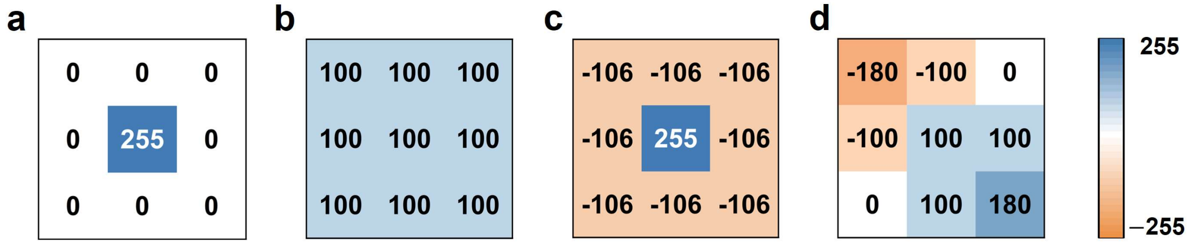

Fig. S10. Convolution kernel matrices: (a) K1: impulse response, (b) K2: smoothing, (c) K3: sharpening and (d) K4: embossing.

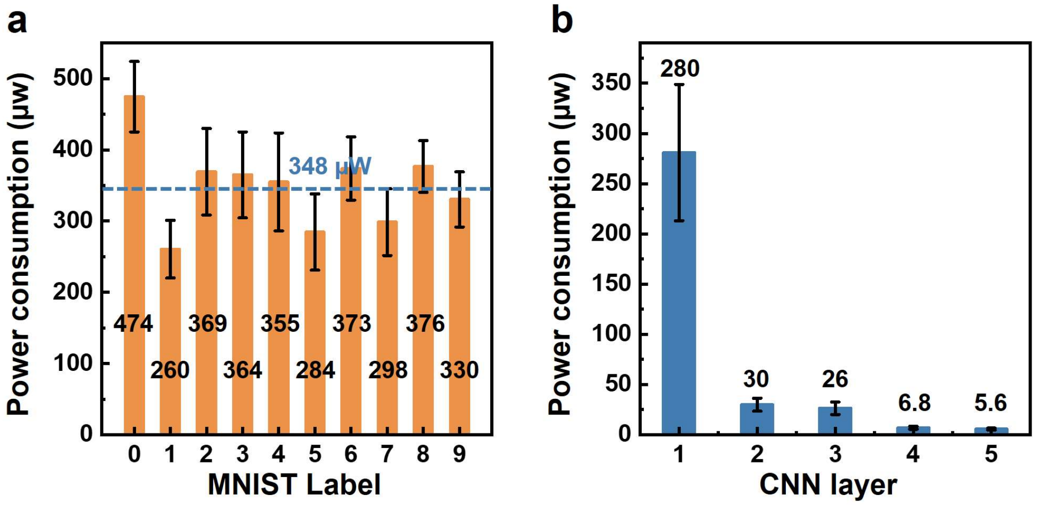

Fig. S11. (a) The power consumption of the CNN during the classification of the ten numerals in the MNIST dataset, based on 200 inferences per numeral. (b) Power consumption of each layer.

Table S1. Physical parameters of capacitors and TFTs making up a 5T3C cell.

Components	Parameters
$C1_{ij}$	2.24 pF
$C2_{ij}$	0.76 pF
$C3_{ij}$	0.69 pF
W/L of an SG TFT	5 μm/5 μm
W/L of a DG TFT	5 μm/5 μm

Table S2. Benchmark metrics of SKCIM system.

Throughput	(32 × 32 × 2) ops / 1 μs × 4 = 8.192 GOP/s
Maximum writing energy / synapse	$1/2 \times 0.76$ pF $\times$ [(5 V)^2 − (1.8 V)^2] = 8.26 pJ
Power	348 μW
Area	988 μm × 1218 μm ≈ 12 mm^2
Performance density	8.192 GOP/s / (12 mm^2 × 4) ≈ 0.17 GOPS/mm^2
Efficiency	8.192 GOP/s / 348 μW ≈ 23.5 TOPS/W
